# Expression of drug targets in primary and matched metastatic renal cell carcinoma tumors

**DOI:** 10.1186/1472-6890-13-3

**Published:** 2013-02-01

**Authors:** Saadia A Aziz, Joshua A Sznol, Adebowale Adeniran, Fabio Parisi, Yuval Kluger, Robert L Camp, Harriet M Kluger

**Affiliations:** 1 Department of Medicine and Yale Cancer Center, 333 Cedar St., WWW213, New Haven, CT, 06520, USA; 2 Department of Pathology, Yale University School of Medicine, New Haven, CT, USA

**Keywords:** Renal cell carcinoma, Targeted therapy, Predictive biomarkers, VEGF

## Abstract

**Background:**

Targeted therapies in renal cell carcinoma can have different effects on primary and metastatic tumors. To pave the way for predictive biomarker development, we assessed differences in expression of targets of currently approved drugs in matched primary and metastatic specimens from 34 patients.

**Methods:**

Four cores from each site were embedded in tissue microarray blocks. Expression of B-Raf, C-Raf, cKIT, FGF-R1, HIF-2α, mTOR, PDGF-Rβ, VEGF-R1, VEGF-R2, VEGF-R3, VEGF, VEGF-B, VEGF-C, VEGF-D, MEK1, and ERK1/2 was studied using a quantitative immunofluorescence method.

**Results:**

No significant differences were observed in global expression levels in primary and metastatic renal cell carcinoma tumors, with the exception of MEK, which had higher expression in metastatic than primary specimens. Similarly, more ki67 positive cells were seen in metastatic specimens. Correlations between marker expression in primary and metastatic specimens were variable, with the lowest correlation seen for FGF-R1 and VEGF-D. There were no significant differences in the degree of heterogeneity in primary versus metastatic tumors.

**Conclusions:**

Expression of most of the studied markers was similar in primary and metastatic renal cell carcinoma tumors, suggesting that predictive biomarker testing for these markers can be conducted on either the primary or metastatic tumors for most markers.

## Background

In recent years, the incidence of renal cell carcinoma (RCC) has increased from 38,000 new cases a year in 2006 to over 64,000 estimated for 2012 [1,2]. This increase is largely due to incidental radiographic identification of renal masses; within this expanding population, RCC diagnoses are shifting towards earlier stage, smaller tumors [3,4]. Despite early detection, the RCC mortality rate remains fairly stable at 13,570 estimated annual deaths [2]. The five-year survival rates for patients with organ-confined disease is >85%, and >50% for patients with regional spread [5], suggesting that tumor biology is variable within the different disease stages.

Surgery followed by surveillance imaging is the standard of care for RCC patients with localized disease. Fine needle aspiration or core needle biopsies are commonly employed for diagnosis of metastatic disease in the 10-50% of these patients with recurring disease. More than 20% of RCC patients present with metastatic disease without having a previously known localized primary tumor [6,7].

RCC is very resistant to standard chemotherapy. Despite advances in biological and immune-based therapies, treatment options for patients with unresectable or metastatic RCC (mRCC) are limited; response rates remain at about 15–44%, and five-year survival under 10% for those with distant metastases [6,8-10]. Immunotherapy once represented the standard treatment for mRCC; interferon-alpha (IFN-α) produces objective responses in 10-15% of patients with a median survival of 12 months, while high-dose Interleukin-2 (IL-2) induces durable remissions in approximately 10% of patients [7,11]. Both are associated with substantial toxicity [12]. Alternative approaches have thus been developed in recent years.

A growing understanding of the pathogenesis of clear cell RCC, the most common histologic subtype, has facilitated development of RCC-targeting therapies. The discovery of Von Hippel-Lindau (VHL) tumor-suppressor gene inactivation and subsequent hypoxia-induced factor (HIF) activation of genes and downstream pathways important to tumor progression have provided the impetus for development of new agents that target angiogenesis and proliferation pathways. A number of drugs that target the vascular endothelial growth factor (VEGF) pathway and downstream signaling molecules have been approved for mRCC. These include the small molecule tyrosine kinase inhibitors (TKIs) sunitinib, sorafenib, pazopanib, and axitinb [10,13-15], the anti-VEGF antibody bevacizumab [16-18], and the mTOR inhibitors temsirolimus and everolimus [19,20]. Other histologic subtypes have different underlying molecular abnormalities, although responses to VEGF and mTOR targeting therapies have been seen in subsets of non-clear cell tumors as well. Although these new agents improve progression free survival and, in some cases, overall survival, none are curative and duration of response is often limited.

Response of primary RCC (as opposed to metastatic deposits) to targeted therapies has not been well studied, however highly sensitive cases are thought to be relatively uncommon. Several groups have reported that pre-surgical targeted therapy can be effective in mRCC patients who have not had their primary tumors removed [21-24]. However, a recent comprehensive study showed minimal to no decrease in the primary tumor diameter in mRCC patients. Patients with a >10% reduction in size of their primary tumors are 2.25 times more likely to have a partial response or stable disease with systemic therapy, indicating that there can be discordance in tumor shrinkage between primary and metastatic tumors in mRCC patients who have not had a nephrectomy [25]. Ongoing clinical trials are assessing the benefits of targeted therapy prior to cytoreductive nephrectomy (NCT00930033, NCT01099423).

At present no predictive biomarkers have been developed to identify patients whose tumors are more likely to respond to any of the currently available therapies. In addition, biomarkers predictive of discordant response between primary and metastatic tumors are lacking. It is therefore necessary to establish patterns of expression of drug targets in tumors in order to attempt to develop predictive tissue based biomarker assays. Given that these drugs exert their effects on the proteome, protein-level predictive biomarkers are a logical place to start. Patterns of expression of drug targets in primary and metastatic RCC tumors have not been previously well demonstrated. Youssif et al. studied 25 matched primary and metastatic samples for correlations between mTOR pathway targets (PTEN, PI3K, p-Akt, phospho-mTOR and p70S6) and found a strong correlation for phospho-mTOR [26]. Here we assess levels of a number of proteins, focusing on targets of currently approved drugs, in four cores from primary tumors and corresponding metastatic deposits. Given the limitations of immunohistochemistry in terms of subjectivity and qualitative assessment of protein expression, we employed a method of quantitative immunofluorescence to measure protein levels. We found that while levels of most markers were not significantly different between primary and metastatic tumors, some markers showed less concordance.

## Methods

### Cohort details and tissue microarray (TMA) construction

Thirty-four patients with archived matched primary and metastatic RCC tumors were identified. Resections were done between 1972 and 2011. TMAs were constructed using cores measuring 0.6 mm in diameter, spaced 0.8 mm apart. Tumors from each of the primary and metastatic tumors were represented by four cores from different areas of the specimen (eight cores in total from each patient) and placed in two TMA blocks. Specimens and clinical information were collected with approval of a Yale University Institutional Review Board.

### Immunofluorescence

Pairs of slides (containing two cores from different areas of each matching primary and metastatic tumor per patient) were stained individually for the target markers; B-Raf, C-Raf, cKIT, FGF-R1 (fibroblast growth factor receptor-1), HIF-2α (hypoxia induced factor-2α), mTOR  (mammalian target of rapamycin), PDGF-Rβ (platelet-derived growth factor receptor-β), VEGF-R1, VEGF-R2, VEGF-R3, VEGF, VEGF-B, VEGF-C, VEGF-D, MEK1 (mitogen activated protein kinase-1), and ERK1/2 (extracellular signal related kinase 1/2). TMAs were also stained for ki67 as a marker of proliferation. Fluorescent staining for our Automated, Quantitative Analysis (AQUA) method was performed as previously described [27,28]. Briefly, slides were incubated with mouse monoclonal anti-human VEGF-D (R&D Systems, Minneapolis, MN, USA), FGF-R1 (QED Bioscience, San Diego, CA, USA), HIF-2α (Novus Biologicals, Littleton, CO, USA), ERK1/2 (Cell Signaling Technologies, Danvers, MA, USA), PDGF-Rβ (Cell Signaling Technologies, Danvers, MA, USA), ki67 (BD Biosciences, San Jose, California, USA), rabbit monoclonal anti-human C-Raf(Upstate/EMD Millipore, Billerica, MA, USA) mTOR (Cell Signaling Technologies, Danvers, MA, USA), MEK1 (Upstate/EMD Millipore, Billerica, MA, USA) and rabbit polyclonal B-Raf, VEGF-R1, VEGF-R2, VEGF-R3 (Santa Cruz Biotechnologies, Santa Cruz, CA, USA), c-Kit (DAKO, Carpinteria, CA, USA) VEGF, VEGF-B (Santa Cruz Biotechnologies, Santa Cruz, CA, USA), VEGF-C (Invitrogen, Carlsbad, CA, USA) overnight at 4°C. Goat anti-mouse (or anti-rabbit) HRP-decorated polymer backbone (Envision, Dako, Carpinteria, CA, USA) was used as a secondary reagent. Slides were incubated with Cyanine5-tyramide (Perkin Elmer, Waltham, MA) in the supplied amplification buffer for 10 min at room temperature. Slides were incubated twice for 7 minutes with 100 mM benzoic hydrazide (B13071, Sigma, St. Louis, MO) and 50 mM hydrogen peroxide in PBS to quench the HRP. To create a tumor mask, slides were incubated with a cocktail of rabbit (for VEGF-D, FGF-R1, HIF-2α, ERK1/2, PDGF-Rβ, ki67) or mouse (for B-Raf, C-Raf, VEGF-R1, VEGF-R2, VEGF-R3, c-Kit, VEGF, VEGF-B, VEGF-C, mTOR and MEK1) anti-cytokeratin (Dako, Carpinteria, CA, USA) 1:100 and HRP-streptavidin(Sigma, St. Louis, MO, USA) for 1 hour at room temperature. HRP-streptavidin binds endogenous biotin which is present is high amounts in RCC and renal tubules. Goat anti-mouse (or anti-rabbit) HRP-decorated polymer backbone (Envision, Dako, Carpinteria, CA, USA) was used as a secondary reagent. Slides were incubated with Cyanine2-tyramide (Perkin Elmer, Waltham, MA, USA) in the supplied amplification buffer for 10 min at room temperature. To create a nuclear mask, TMAs were incubated with 4, 6-diamidine-2-phenylindole (DAPI) at a concentration of 1:500 in 0.3% BSA in TBS. Coverslips were mounted with ProLong Gold antifade reagent with 4, 6-diamidine-2-phenylindole (DAPI) (Invitrogen, Carlsbad, CA).

### Automated image acquisition and analysis

Images were acquired and analyzed using previously described algorithms [29]. Briefly, monochromatic, high-resolution (1024 × 1024 pixel) images were obtained of each histospot. Tumor was distinguished from stroma by the cytokeratin/streptavidin signal. Cell surface coalescence of cytokeratin was used to localize membranes and DAPI to identify nuclei. The target signal (B-Raf, C-Raf, cKIT, FGF-R1, HIF-2α, mTOR, PDGF-Rβ, VEGF-R1, VEGF-R2, VEGF-R3, VEGF, VEGF-B, VEGF-C, VEGF-D, MEK1, and ERK1/2) from the pixels within the cytoplasm was normalized to area of tumor mask and scored on a scale of 0–255 (the AQUA score). Ki-67 positivity was calculated as a percentage of tumor cells. Histospots were excluded if the tumor mask represented <5% of the histospot area or if there was anomalous staining (lacking DAPI or cytokeratin, or necrotic tissue).

### Statistical analysis

Statview and JMP 5.0 software were used (SAS Institute, Cary, NC). AQUA scores for replicate tumor cores were averaged. Associations between continuous AQUA scores of the target and clinical and pathological parameters were assessed using ANOVA. Correlations between the AQUA scores of matched primary and metastatic histospots were calculated by the log rank method. Intratumor heterogeneity was assessed by Pearson linear regression.

## Results

Patient characteristics are summarized in Table 1. Histological subtypes included clear cell (ccRCC) (91.2%) and mixed histology (8.8%). Age at diagnosis was 17–72 years (median-56). Performance status, LDH, hemoglobin and calcium levels were not available.

**Table 1:** Patient characteristics

**Patient**	**Progression time: time to metastasis (months)**	**Age at primary**	**Tumor size (cm)**	**Histology**	**Fuhrman grade**	**Gender**	**Metastasis location**
1	6	51	5	Clear Cell	3	M	Lung
2	72	64	3.2	Clear Cell	3	M	Lung
3	6	50	10	Clear Cell	3	M	Abdominal Wall
4	12	45	7	Clear Cell	3	M	Skin
5	12	70	4	Clear Cell	2	M	Lung
6	6	58	3	Clear Cell	2	F	Colon
7	24	17	13	Clear Cell	3	F	Lung
8	36	66	4	Clear Cell	2	M	Lung
9	24	64	10.7	Clear Cell	3	M	Liver
10	48	69	6	Clear Cell	3	F	Skin
11	36	61	8	Clear Cell	3	F	Lung
12	24	61	8	Clear Cell	3	F	Liver
13	36	68	4.5	Clear Cell	1	M	Bone
14	6	56	11	Clear Cell	3	F	Pituitary
15	120	47	8	Clear Cell	3	M	Testes
16	24	54	6.5	Clear Cell	2	F	Soft Tissue
17	48	62	10.5	Clear Cell	3	M	Bone
18	156	56	3.5	Clear Cell	3	M	Lung
19	108	59	8.5	Clear Cell	2	M	Lung
20	12	28	4	Mixed	3	M	Lymph Node
21	6	54	Unknown	Clear Cell	2	M	Lung
22	12	40	3.7	Mixed	4	M	Lung
23	6	64	3.5	Clear Cell	2	M	Bone
24	84	46	14.5	Clear Cell	2	F	Lung
25	36	55	8.5	Clear Cell	2	M	Lung
26	36	66	6.5	Clear Cell	2	M	Adrenal
27	132	72	5	Clear Cell	2	F	Adrenal
28	12	72	6	Mixed	3	F	Soft Tissue
29	6	52	3.5	Clear Cell	3	F	Bone
30	12	50	12	Clear Cell	2	F	Skin
31	6	33	3.5	Clear Cell	2	M	Bone
32	36	69	10.5	Clear Cell	3	F	Bone
33	12	62	6	Clear Cell	3	M	Bone
34	36	47	8	Clear Cell	3	F	Lung

Each lot of antibodies was subjected to immunoblotting to verify presence of a single dominant band of the appropriate size (not shown). We note that a number of antibodies to VEGF-R2 are commercially available, and a recent publication demonstrated higher specificity of the 55B11 antibody (Cell Signaling Technologies, Danvers, MA) than the A-3 antibody (Santa Cruz Biotechnologies, Inc, Santa Cruz, CA) [30]. However, in our hands, using quantitative immunofluorescence, we found superior correlations between redundant spots, membrane-specific staining and better reproducibility of results with the A-3 antibody. Western blotting for the A-3 antibody showed a single band at the associated protein size. The discrepancy between our findings and those of Molhoek et al. [30] might be due to batch-to-batch variability and the quantitative staining method used here. Information on antibodies used is given in the Additional file 1: Table S1.

**Figure 1:**
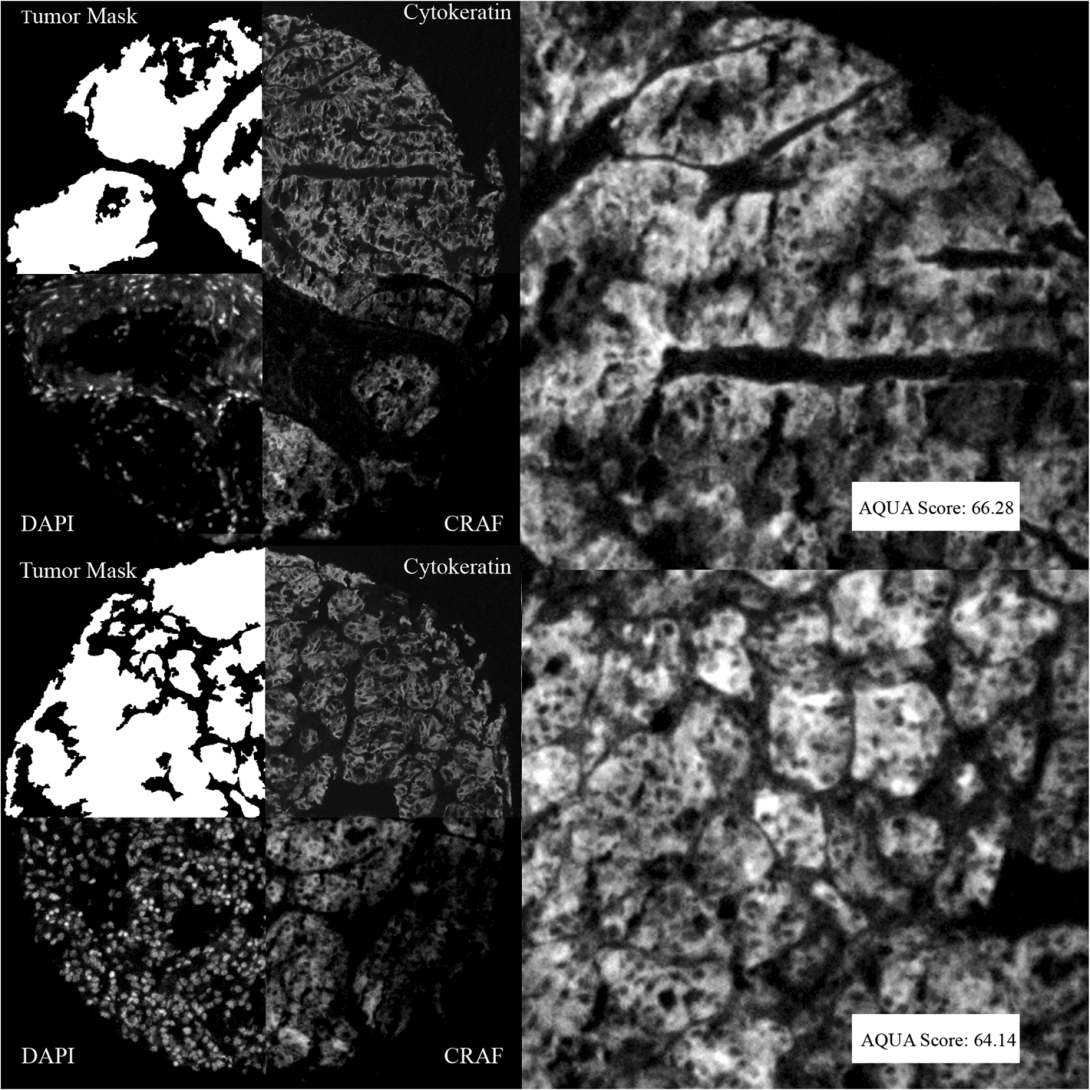
**Example of Automated Quantitative Analysis (AQUA) staining for CRAF in matched primary (upper panels) and metastatic (lower panels) specimens from a single patient: We used anti-cytokeratin antibodies to create a cytoplasmic compartment (two upper right quadrants).** A tumor mask was made by filling in holes (upper left quadrants). 4’, 6-diamidino-2-phenylindole (DAPI) defines the nuclear compartment within the tumor mask (left lower quadrants). CRAF expression is measured within the cytoplasmic compartments, within the tumor mask (lower right quadrants), and each clinical case is assigned a score based on pixel intensity per unit area within the tumor mask. The upper panel shows an example of a histospot from a primary specimen and the lower shows the corresponding metastatic tumor. CRAF staining was strong and similar in both specimens.

Figure 1a-d shows an example of C-Raf expression in corresponding primary and metastatic tissues of one patient. AQUA scores for the primary and metastatic tumors for this patient were 66.28 and 64.14, respectively. To assess intratumor heterogeneity, four distinct cores from both the primary and metastatic sites were used to evaluate expression of all the markers. Subsequently, scores from corresponding cores were averaged to obtain a single concatenated score for each tumor for each marker.

Global distribution of scores in primary and metastatic spots was not significantly different for any of the markers with the exception of MEK1. The mean AQUA scores and the differences between primary and metastatic tumors by paired t-tests are shown in Table 2. No significant differences were found between expression in primary and metastatic specimens for B-Raf, C-Raf, cKIT, FGF-R1, HIF-2α, mTOR, PDGF-Rβ, VEGF-R1, VEGF-R2, VEGF-R3, VEGF, VEGF-B, VEGF-C, VEGF-D, and ERK1/2. Expression of MEK1 was somewhat higher in metastatic than primary tumors (P = 0.002). The p-value of 0.002 for MEK is below the Bonferroni adjusted p-value (0.003) for the 16 markers analyzed at an alpha of 0.05. As MEK is an important component of the major intracellular proliferation signaling pathway, we looked at percentage of cells in tumors with ki67 staining, and found that ki67 positivity was significantly higher (P = 0.0006) in metastatic than primary tumors.

**Table 2:** Tyrosine kinase and mTOR inhibitor target expression in primary and metastatic RCC samples

**Target**	**AQUA scores, primary specimens (mean ± SD)**	**AQUA scores, metastatic specimens (mean ± SD)**	***t*-statistic**	***p*-value**
B-Raf	32.1 ± 8.9	31.2 ± 10.2	−0.417	0.678
C-Raf	26.7 ± 2.1	28.4 ± 2.5	0.519	0.605
cKit	23.3 ± 1.3	23.4 ± 1.3	0.079	0.937
FGF-R1	29.1 ± 1.1	29.9 ± 0.9	0.579	0.564
HIF-2α	58.2 ± 1.9	58.2 ± 2.0	0.014	0.989
mTOR	18.8 ± 1.4	22.4 ± 1.8	1.637	0.106
PDGF-Rβ	24.7 ± 0.9	27.9 ± 1.5	1.879	0.064
VEGF-R1	22.6 ± 0.9	23.9 ± 1.2	0.34	0.735
VEGF-R2	36.5 ± 2.4	33.5 ± 1.7	−1.294	0.200
VEGF-R3	45.6 ± 2.0	46.5 ± 1.6	0.371	0.712
VEGF	24.7 ± 1.0	26.0 ± 1.1	0.907	0.367
VEGF-B	11.2 ± 0.6	11.8 ± 0.9	0.486	0.628
VEGF-C	17.0 ± 1.3	14.3 ± 1.2	−1.544	0.127
VEGF-D	35.0 ± 1.2	36.8 ± 1.3	1.018	0.312
MEK1	37.5 ± 2.5	50.3 ± 3.1	3.183	0.002
ERK1/2	17.0 ± 1.1	18.6 ± 1.7	0.802	0.425

Given that archival tissue is often available from either the primary or the metastatic site, but not both, we determined the associations between marker expression in paired primary and metastatic samples using the Pearson correlation test, as displayed in Table 3. Levels correlated well between primary and metastatic specimens for most markers, with the poorest correlation seen for FGF-R1 and VEGF-D (0.15 and 0.28, respectively).

**Table 3:** Correlations between paired primary and metastatic samples

**Marker**	**R value**
B-Raf	0.63
c-Kit	0.59
C-Raf	0.83
MEK1	0.53
FGF-R1	0.15
HIF-2α	0.77
mTOR	0.66
PDGF-Rβ	0.65
VEGFR-1	0.69
VEGFR-2	0.84
VEGFR-3	0.53
VEGF	0.49
VEGF-B	0.66
VEGF-C	0.81
VEGF-D	0.28
ERK 1/2	0.36

**Figure 2:**
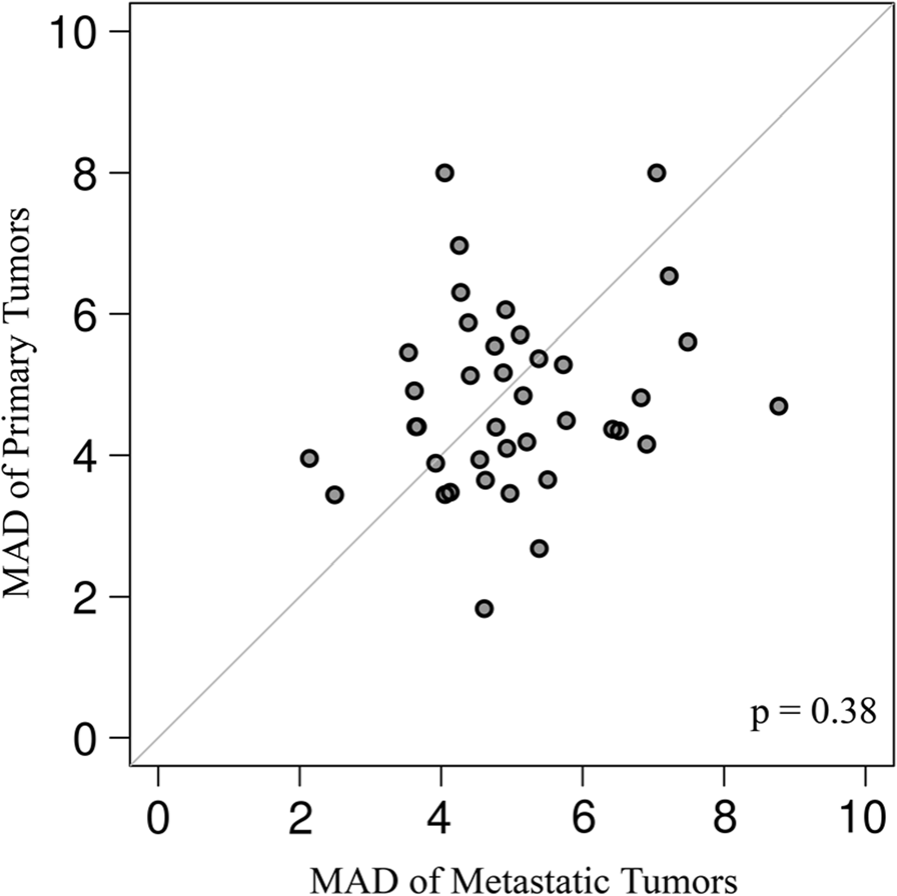
**Comparison between heterogeneity within primary and metastatic specimens, estimated using a composite median absolute deviation (MAD) across all the markers: Each patient is represented by a dot.** Dots above the diagonal represent patients with larger heterogeneity in the primary tumors, while dots below the diagonal represent greater heterogeneity in the corresponding metastatic tumors. The central diagonal grey line represents identical heterogeneity in primary and metastatic tumors. The Wilcoxon paired, two-sided signed rank test shows no significant difference between the heterogeneities of the primary and matched metastatic tumors (p = 0.38).

To determine whether there were differences in intra-tumor heterogeneity in primary and metastatic specimens, we used the four measurements from each tumor. Each core is represented by a vector of measurements of all markers, denoted as the core vector. For each patient we computed the median core vector and measured its L1 distances from the corresponding four core vectors. We defined the composite median absolute deviation (MAD) as the median of these four L1 distances, and used it as a proxy for estimating intra-tumor heterogeneity. For each patient, the composite MAD is computed separately for his/her primary and metastatic tumors. Using the Wilcoxon paired, two-sided signed rank test, we found no significant differences in heterogeneity between primary and metastatic tumors (P = 0.38), as shown in Figure 2. 

## Discussion

Molecular targeted therapies that inhibit members of the VEGF pathway and mTOR are now widely used for the treatment of metastatic RCC. At present, no predictive biomarkers have been established for this class of drugs. Given that these agents inhibit this pathway at the protein level, target protein expression might be associated with response to therapy. Many metastatic RCC patients have either primary or metastatic tumor tissue (but not both) available for analysis, and our purpose was therefore to determine differences in expression of these drug targets in matched primary and metastatic specimens.

Target expression levels were not globally different between primary and metastatic tumors, with the exception of MEK1, which was higher in metastatic specimens. Given that MEK1 is a key component of the major intracellular proliferation signal transduction pathway, we studied ki67 expression in primary and metastatic samples and found that the percentage of ki67 positive cells was also significantly higher in the metastases. This is consistent with the commonly seen clinical picture in which the primary tumor grows over years, yet the course for metastatic disease patients is often much shorter due to rapid metastatic tumor growth.

Our data indicate that our ability to predict expression in primary specimens based on measurements from a corresponding metastasis and vice-versa is marker dependent. The intra-patient correlations were variable across markers, with the worst correlation shown for VEGF-D and FGF-R1, while other makers such as C-Raf, VEGF-R2 and cKIT demonstrated excellent correlations between levels in primary and metastatic specimens. These findings are consistent with the only other similar published study of which we are aware in which mTOR pathway members were assessed for concordance between primary and metastatic sites using standard immunohistochemistry (IHC) [26]. Levels of phosphorylated mTOR were similar in primary and metastatic sites, while levels of PI3K, p-Akt, PTEN and p70S6 had much weaker intra-patient correlations. We elected not to study levels of phosphorylated proteins in our study, as many of these specimens were old and fixation times were not uniform. Phosphatase activity, therefore, cannot be accounted for in our samples. Use of nominal IHC scores might make similarities in expression less apparent than use of continuous AQUA scores.

To date, none of the approved drugs for mRCC has an associated companion diagnostic biomarker test. A number of initial attempts have been made at developing predictive biomarkers that are primarily centered around VHL pathway markers, such as VHL mutations, HIF levels, VEGF isoforms and VEGF receptor levels. Phase II trials of axitinib or temsirolimus revealed no association between VHL mutational status and response to therapy [10,31]. Higher levels of pS6 and p-AKT in pre-treatment tissues were associated with response to temsirolimus, but no significant difference was found between primary and metastatic tissues. In addition, a number of studies have looked at VHL loss and response to immunotherapy [31,32]. A study of 123 patients with clear cell RCC showed that patients with wild-type VHL had a lower likelihood of responding to VEGF-pathway targeted therapies than patients with VHL mutations or VHL loss by hypermethylation [33]. This finding, however, requires further validation.

Small studies have suggested a potential role for VEGF and soluble forms of the VEGF receptors as predictors of response to VEGF pathway targeting therapies and cytokine therapies. Sabatino et al. [34] measured serum protein levels using multiplex protein arrays, and showed that high pretreatment levels of VEGF and fibronectin were negative predictors of response to IL-2. Trials using bevacizumab with interferon or sorafenib showed no predictive value of baseline VEGF levels in patients [13,17]. In a phase II trial, Hutson et al. found that decreased expression of soluble VEGFR-2 correlated with tumor response to pazopanib [35]. Similarly, in a phase II trial of sunitinib, increases in soluble VEGFR-2, VEGFR-3, and VEGF at day 28 were associated with a greater likelihood of response [36].

Clinical observations of discordance in response of primary and metastatic tumors suggest possible differences in biology. Alternatively, differences in response could be due to variable tumor microenvironment in the primary and metastatic sites. Primary RCC tumors do not seem to respond as well as RCC metastatic sites to VEGF pathway targeted therapies [25]. Studies evaluating targeted therapies in RCC for their preoperative potential to reduce the size of primary tumors with the hope of making them more resectable are ongoing. Prior studies evaluating sunitinib and/or sorafenib in patients with localized and metastatic RCC disease concluded that these agents might be useful in reducing primary tumor burden [21-24,37]. A phase II study of presurgical sunitinib resulted in only 1 partial primary tumor response, while another study concluded that preoperative sunitinib can be effective for cytoreduction. Eighty percent of patients demonstrated variable primary tumor shrinkage, with a median of 1.6 cm (range 0.4- 5.1) [38,39]. A larger study of 168 mRCC patients who received targeted therapy with their primary tumors in situ found noted negligible decreases in the size of the primary tumors [25]. Although contradictory, these studies do suggest discordant responses to drugs in primary and metastatic tumors. Additional trials are needed to determine whether any of the biomarkers studied here is predictive of response to VEGF pathway targeting therapies, and when discordant tumor shrinkage is observed, whether it can be explained by differences in biomarker expression in primary and metastatic samples.

Biomarker studies related to evolving biomarkers and experimental drugs are being done by our group and others. The clinical relevance of HIF-1α and HIF-2α are being studied as the hypoxia induced pathway is regularly aberrant in RCC. HIF-1α has been shown to be expressed in most RCC tumors while HIF-2α is relatively absent in early tumors, but is highly expressed in metastatic tumors [40,41]. B7-H1 is another target that is being heavily explored, with multiple clinical trials of B7-H1 targeting ongoing. A study by Thompson et. al in primary and metastatic RCC showed high B7-H1 expression is associated with a poor prognosis. Although only 1 patient was represented in both cohorts, more metastatic specimens had high B7-H1 expression than primary specimens (54.3% versus 44.4%, respectively) [42]. Tumor suppressor gene p53 was significantly higher in primary tumors versus metastatic tumors in a study by Zigeuner et. al (22.8% versus 51.8%), however the specimens were not matched [43]. In a study of mTOR and hypoxia-induced pathway members including 135 primary RCC and 41 unrelated metastasis, differential global patterns of expression were measured. Levels of p-AKT, p-S6, 4EBP1, and c-myc were higher in metastatic lesions compared to both primary and benign tissues [44].

The tumors studied here exhibited variable intratumor heterogeneity in the four tumor cores. The degree of heterogeneity is not significantly different in primary and metastatic samples. Although our study evaluates protein expression, recent DNA sequencing studies have shown intratumor heterogeneity in primary renal cell carcinoma. The majority of somatic mutations (over 60%) were not present throughout the tumor in the 4 samples examined. Moreover, DNA signatures of both good and poor prognosis were detected in different regions of the same tumor. The authors suggest that intratumor heterogeneity is the cause of lack of reproducible predictive biomarkers [45]. Utilizing single-cell exome sequencing in a single patient, Xu et al. demonstrated that there was no dominant clone throughout the tumor, and similarly demonstrated heterogeneity at the DNA level. This may provide insight into the observed heterogeneity in this study [46].

## Conclusion

Our studies showed good concordance between primary and metastatic samples for most of the markers studied. The biomarkers with the least concordance were FGF-R1 and VEGF-D. The discordance in levels of VEGF-D might be due to the fact that this is a secreted protein, and levels of FGF-R1 might be more influenced by the tumor microenvironment than the other markers studied. Conversely, other biomarkers showed excellent concordance between primary and metastatic samples. As predictive biomarkers are developed, careful studies are needed to define concordance versus discordance for individual biomarkers in order to determine whether primary specimen measurements can be used as surrogates for metastatic specimens and vice-versa.

## Competing interests

R.L. Camp is a co-founder, stockholder and consultant for a company called HistoRx that has licensed the technology for automated tissue analysis used in this study.

## Authors’ contributions

SA drafted the manuscript, acquired the data, and participated in the analysis of data. JS helped with the acquisition of data and the analysis. AA performed the pathology review of specimens. FP and YK conducted statistical and computational analysis for this study. RC designed the tissue microarrays and performed pathology review. HK designed and coordinated the study and participated in the drafting of this manuscript. All authors read and approved the final manuscript.

## Pre-publication history

The pre-publication history for this paper can be accessed here:

http://www.biomedcentral.com/1472-6890/13/3/prepub

## Supplementary Material

Additional file 1: Table S1 List of Antibodies. Click here for file

## References

[B1] Jemal A Siegel R Ward E Murray T Xu J Smigal C Thun MJ Cancer statistics, 2006 CA Cancer J Clin 2006 56 106 130 10.3322/canjclin.56.2.106 16514137

[B2] Siegel R Naishadham D Jemal A Cancer statistics, 2012 CA Cancer J Clin 2012 62 10 29 10.3322/caac.20138 22237781

[B3] Kane CJ Mallin K Ritchey J Cooperberg MR Carroll PR Renal cell cancer stage migration: analysis of the National Cancer Data Base Cancer 2008 113 78 83 10.1002/cncr.23518 18491376

[B4] Sanchez-Martin FM Millan-Rodriguez F Urdaneta-Pignalosa G Rubio-Briones J Villavicencio-Mavrich H Small renal masses: incidental diagnosis, clinical symptoms, and prognostic factors Adv Urol 2008 310694 19165347 10.1155/2008/310694 PMC2629071

[B5] Oudard S George D Medioni J Motzer R Treatment options in renal cell carcinoma: past, present and future Ann Oncol 2007 18 Suppl 10 x25 x31 17761720 10.1093/annonc/mdm411

[B6] Lam JS Leppert JT Belldegrun AS Figlin RA Novel approaches in the therapy of metastatic renal cell carcinoma World J Urol 2005 23 202 212 10.1007/s00345-004-0466-0 15812574

[B7] Motzer RJ Bander NH Nanus DM Renal-cell carcinoma N Engl J Med 1996 335 865 875 10.1056/NEJM199609193351207 8778606

[B8] Figlin RA Renal cell carcinoma: management of advanced disease J Urol 1999 161 381 386 discussion 386–387 10.1016/S0022-5347(01)61897-4 9915408

[B9] Bukowski RM Prognostic factors for survival in metastatic renal cell carcinoma: update 2008 Cancer 2009 115 2273 2281 10.1002/cncr.24226 19402065

[B10] Rini BI Escudier B Tomczak P Kaprin A Szczylik C Hutson TE Michaelson MD Gorbunova VA Gore ME Rusakov IG Comparative effectiveness of axitinib versus sorafenib in advanced renal cell carcinoma (AXIS): a randomised phase 3 trial Lancet 2011 378 1931 1939 10.1016/S0140-6736(11)61613-9 22056247

[B11] Fyfe G Fisher RI Rosenberg SA Sznol M Parkinson DR Louie AC Results of treatment of 255 patients with metastatic renal cell carcinoma who received high-dose recombinant interleukin-2 therapy J Clin Oncol 1995 13 688 696 7884429 10.1200/JCO.1995.13.3.688

[B12] Facchini G Perri F Caraglia M Pisano C Striano S Marra L Fiore F Aprea P Pignata S Iaffaioli RV New treatment approaches in renal cell carcinoma Anticancer Drugs 2009 20 893 900 10.1097/CAD.0b013e32833123d4 19752718

[B13] Escudier B Eisen T Stadler WM Szczylik C Oudard S Staehler M Negrier S Chevreau C Desai AA Rolland F Sorafenib for treatment of renal cell carcinoma: Final efficacy and safety results of the phase III treatment approaches in renal cancer global evaluation trial J Clin Oncol 2009 27 3312 3318 10.1200/JCO.2008.19.5511 19451442

[B14] Motzer RJ Hutson TE Tomczak P Michaelson MD Bukowski RM Rixe O Oudard S Negrier S Szczylik C Kim ST Sunitinib versus interferon alfa in metastatic renal-cell carcinoma N Engl J Med 2007 356 115 124 10.1056/NEJMoa065044 17215529

[B15] Sternberg CN Davis ID Mardiak J Szczylik C Lee E Wagstaff J Barrios CH Salman P Gladkov OA Kavina A Pazopanib in locally advanced or metastatic renal cell carcinoma: results of a randomized phase III trial J Clin Oncol 2010 28 1061 1068 10.1200/JCO.2009.23.9764 20100962

[B16] Yang JC Haworth L Sherry RM Hwu P Schwartzentruber DJ Topalian SL Steinberg SM Chen HX Rosenberg SA A randomized trial of bevacizumab, an anti-vascular endothelial growth factor antibody, for metastatic renal cancer N Engl J Med 2003 349 427 434 10.1056/NEJMoa021491 12890841 PMC2275324

[B17] Escudier B Bellmunt J Negrier S Bajetta E Melichar B Bracarda S Ravaud A Golding S Jethwa S Sneller V Phase III trial of bevacizumab plus interferon alfa-2a in patients with metastatic renal cell carcinoma (AVOREN): final analysis of overall survival J Clin Oncol 2010 28 2144 2150 10.1200/JCO.2009.26.7849 20368553

[B18] Rini BI Halabi S Rosenberg JE Stadler WM Vaena DA Archer L Atkins JN Picus J Czaykowski P Dutcher J Small EJ Phase III trial of bevacizumab plus interferon alfa versus interferon alfa monotherapy in patients with metastatic renal cell carcinoma: final results of CALGB 90206 J Clin Oncol 2010 28 2137 2143 10.1200/JCO.2009.26.5561 20368558 PMC2860433

[B19] Hudes G Carducci M Tomczak P Dutcher J Figlin R Kapoor A Staroslawska E Sosman J McDermott D Bodrogi I Temsirolimus, interferon alfa, or both for advanced renal-cell carcinoma N Engl J Med 2007 356 2271 2281 10.1056/NEJMoa066838 17538086

[B20] Motzer RJ Escudier B Oudard S Hutson TE Porta C Bracarda S Grunwald V Thompson JA Figlin RA Hollaender N Efficacy of everolimus in advanced renal cell carcinoma: a double-blind, randomised, placebo-controlled phase III trial Lancet 2008 372 449 456 10.1016/S0140-6736(08)61039-9 18653228

[B21] Amin C Wallen E Pruthi RS Calvo BF Godley PA Rathmell WK Preoperative tyrosine kinase inhibition as an adjunct to debulking nephrectomy Urology 2008 72 864 868 10.1016/j.urology.2008.01.088 18684493

[B22] Shuch B Riggs SB LaRochelle JC Kabbinavar FF Avakian R Pantuck AJ Patard JJ Belldegrun AS Neoadjuvant targeted therapy and advanced kidney cancer: observations and implications for a new treatment paradigm BJU Int 2008 102 692 696 10.1111/j.1464-410X.2008.07660.x 18410444

[B23] Thomas AA Rini BI Lane BR Garcia J Dreicer R Klein EA Novick AC Campbell SC Response of the primary tumor to neoadjuvant sunitinib in patients with advanced renal cell carcinoma J Urol 2009 181 518 523 discussion 523 19100579 10.1016/j.juro.2008.10.001

[B24] van der Veldt AA Meijerink MR van den Eertwegh AJ Bex A de Gast G Haanen JB Boven E Sunitinib for treatment of advanced renal cell cancer: primary tumor response Clin Cancer Res 2008 14 2431 2436 10.1158/1078-0432.CCR-07-4089 18413834

[B25] Abel EJ Culp SH Tannir NM Matin SF Tamboli P Jonasch E Wood CG Primary tumor response to targeted agents in patients with metastatic renal cell carcinoma Eur Urol 2011 59 10 15 10.1016/j.eururo.2010.09.034 20952123 PMC4378833

[B26] Abou Youssif T Fahmy MA Koumakpayi IH Ayala F Al Marzooqi S Chen G Tamboli P Squire J Tanguay S Sircar K The mammalian target of rapamycin pathway is widely activated without PTEN deletion in renal cell carcinoma metastases Cancer 2011 117 290 300 10.1002/cncr.25402 20830770

[B27] Camp RL Chung GG Rimm DL Automated subcellular localization and quantification of protein expression in tissue microarrays Nat Med 2002 8 1323 1327 10.1038/nm791 12389040

[B28] Aziz SA Davies M Pick E Zito C Jilaveanu L Camp RL Rimm DL Kluger Y Kluger HM Phosphatidylinositol-3-kinase as a therapeutic target in melanoma Clin Cancer Res 2009 15 3029 3036 10.1158/1078-0432.CCR-08-2768 19383818 PMC4431617

[B29] Kluger HM Siddiqui SF Angeletti C Sznol M Kelly WK Molinaro AM Camp RL Classification of renal cell carcinoma based on expression of VEGF and VEGF receptors in both tumor cells and endothelial cells Lab Invest 2008 88 962 972 10.1038/labinvest.2008.65 18626467

[B30] Molhoek KR Erdag G Rasamny JK Murphy C Deacon D Patterson JW Slingluff CL Jr Brautigan DL VEGFR-2 expression in human melanoma: revised assessment Int J Cancer 2011 129 2807 2815 10.1002/ijc.25963 21544800 PMC3205910

[B31] Cho D Signoretti S Dabora S Regan M Seeley A Mariotti M Youmans A Polivy A Mandato L McDermott D Potential histologic and molecular predictors of response to temsirolimus in patients with advanced renal cell carcinoma Clin Genitourin Cancer 2007 5 379 385 10.3816/CGC.2007.n.020 17956710

[B32] Lam JS Leppert JT Figlin RA Belldegrun AS Role of molecular markers in the diagnosis and therapy of renal cell carcinoma Urology 2005 66 1 9 16194700 10.1016/j.urology.2005.06.112

[B33] Choueiri TK Vaziri SA Jaeger E Elson P Wood L Bhalla IP Small EJ Weinberg V Sein N Simko J von Hippel-Lindau gene status and response to vascular endothelial growth factor targeted therapy for metastatic clear cell renal cell carcinoma J Urol 2008 180 860 865 discussion 865–866 10.1016/j.juro.2008.05.015 18635227

[B34] Sabatino M Kim-Schulze S Panelli MC Stroncek D Wang E Taback B Kim DW Deraffele G Pos Z Marincola FM Kaufman HL Serum vascular endothelial growth factor and fibronectin predict clinical response to high-dose interleukin-2 therapy J Clin Oncol 2009 27 2645 2652 10.1200/JCO.2008.19.1106 19364969 PMC2689845

[B35] Hutson TE Davis ID Machiels JP De Souza PL Rottey S Hong BF Epstein RJ Baker KL McCann L Crofts T Efficacy and safety of pazopanib in patients with metastatic renal cell carcinoma J Clin Oncol 2010 28 475 480 10.1200/JCO.2008.21.6994 20008644

[B36] Hutson TE Sunitinib (SUTENT) for the treatment of metastatic renal cell carcinoma Expert Rev Anticancer Ther 2008 8 1723 1731 10.1586/14737140.8.11.1723 18928373

[B37] Cowey CL Amin C Pruthi RS Wallen EM Nielsen ME Grigson G Watkins C Nance KV Crane J Jalkut M Neoadjuvant clinical trial with sorafenib for patients with stage II or higher renal cell carcinoma J Clin Oncol 2010 28 1502 1507 10.1200/JCO.2009.24.7759 20159822 PMC4525128

[B38] Griffioen AW Mans LA de Graaf AM Nowak-Sliwinska P de Hoog CL de Jong TA Vyth-Dreese FA van Beijnum JR Bex A Jonasch E Rapid angiogenesis onset after discontinuation of sunitinib treatment of renal cell carcinoma patients Clin Cancer Res 2012 18 3961 3971 10.1158/1078-0432.CCR-12-0002 22573349 PMC4015630

[B39] Rini BI Garcia J Elson P Wood L Shah S Stephenson A Salem M Gong M Fergany A Rabets J The effect of sunitinib on primary renal cell carcinoma and facilitation of subsequent surgery J Urol 2012 187 1548 1554 10.1016/j.juro.2011.12.075 22425095

[B40] Raval RR Lau KW Tran MG Sowter HM Mandriota SJ Li JL Pugh CW Maxwell PH Harris AL Ratcliffe PJ Contrasting properties of hypoxia-inducible factor 1 (HIF-1) and HIF-2 in von Hippel-Lindau-associated renal cell carcinoma Mol Cell Biol 2005 25 5675 5686 10.1128/MCB.25.13.5675-5686.2005 15964822 PMC1157001

[B41] Mandriota SJ Turner KJ Davies DR Murray PG Morgan NV Sowter HM Wykoff CC Maher ER Harris AL Ratcliffe PJ Maxwell PH HIF activation identifies early lesions in VHL kidneys: evidence for site-specific tumor suppressor function in the nephron Cancer Cell 2002 1 459 468 10.1016/S1535-6108(02)00071-5 12124175

[B42] Thompson RH Gillett MD Cheville JC Lohse CM Dong H Webster WS Chen L Zincke H Blute ML Leibovich BC Kwon ED Costimulatory molecule B7-H1 in primary and metastatic clear cell renal cell carcinoma Cancer 2005 104 2084 2091 10.1002/cncr.21470 16208700

[B43] Zigeuner R Ratschek M Rehak P Schips L Langner C Value of p53 as a prognostic marker in histologic subtypes of renal cell carcinoma: a systematic analysis of primary and metastatic tumor tissue Urology 2004 63 651 655 10.1016/j.urology.2003.11.011 15072872

[B44] Schultz L Chaux A Albadine R Hicks J Kim JJ De Marzo AM Allaf ME Carducci MA Rodriguez R Hammers HJ Immunoexpression status and prognostic value of mTOR and hypoxia-induced pathway members in primary and metastatic clear cell renal cell carcinomas Am J Surg Pathol 2011 35 1549 1556 10.1097/PAS.0b013e31822895e5 21881486 PMC3505672

[B45] Gerlinger M Rowan AJ Horswell S Larkin J Endesfelder D Gronroos E Martinez P Matthews N Stewart A Tarpey P Intratumor heterogeneity and branched evolution revealed by multiregion sequencing N Engl J Med 2012 366 883 892 10.1056/NEJMoa1113205 22397650 PMC4878653

[B46] Xu X Hou Y Yin X Bao L Tang A Song L Li F Tsang S Wu K Wu H Single-cell exome sequencing reveals single-nucleotide mutation characteristics of a kidney tumor Cell 2012 148 886 895 10.1016/j.cell.2012.02.025 22385958 PMC7458411

