# Supplementary material for: Expression of drug targets in primary and matched metastatic renal cell carcinoma tumors
Source: BMC Clin Pathol. 2013 Feb 1;13:3. doi: 10.1186/1472-6890-13-3 (PMC3575219; doi:10.1186/1472-6890-13-3)
Supplement: Additional file 1: Table S1 — List of Antibodies. [file 1472-6890-13-3-S1.doc]

**Supplemental Table 1. List of Antibodies**.

| **Target Name** | **Source** | **Concentration**  **IHC** |
| --- | --- | --- |
| **B-Raf** | **Santa Cruz**  **(rabbit polyclonal) C-19** | **1:100** |
| **C-Raf** | **Upstate**  **(rabbit monoclonal) 04-412** | **1:100** |
| **ERK1/2** | **Cell Signaling**  **(mouse monoclonal) L34F12** | **1:3000** |
| **VEGF-R1** | **Santa Cruz**  **(rabbit polyclonal) C-17** | **1:200** |
| **VEGF-R2** | **Santa Cruz**  **(mouse monoclonal) A-3** | **1:200** |
| **VEGF-R3** | **Santa Cruz**  **(rabbit polyclonal) C-20** | **1:500** |
| **FGF-R1** | **QED Bioscience**  **(mouse monoclonal) M5G10** | **1:400** |
| **PDGF-Rβ** | **BD Transduction Lab.**  **(mouse monoclonal) C-28** | **1:1000** |
| **CD117, c-Kit** | **Dako (rabbit polyclonal)**  **A4502** | **1:50** |
| **mTOR** | **Cell Signaling**  **(rabbit monoclonal)7C10** | **1:40** |
| **VEGF** | **Santa Cruz (rabbit polyclonal)**  **A-20** | **1:500** |
| **VEGF-B** | **Santa Cruz (rabbit polyclonal)**  **H-70** | **1:900** |
| **VEGF-C** | **Invitrogen (rabbit polyclonal)**  **Z-CVC7** | **1:800** |
| **VEGF-D** | **R&D Systems(mouse monoclonal) 78923** | **1:600** |
| **HIF-2α** | **Novus Biologicals (mouse monoclonal) ep190b** | **1:200** |
